# Psychometric properties of the Persian Gaming Disorder Test and relationships with psychological distress and insomnia in adolescents

**DOI:** 10.1186/s40359-023-01368-z

**Published:** 2023-10-10

**Authors:** Chung-Ying Lin, Marc N. Potenza, Halley M. Pontes, Amir H. Pakpour

**Affiliations:** 1 Institute of Allied Health Sciences, College of Medicine, National Cheng Kung University, Tainan, 701 Taiwan; 2 Biostatistics Consulting Center, College of Medicine, National Cheng Kung University Hospital, National Cheng Kung University, Tainan, 701 Taiwan; 3 Departments of Psychiatry and Neuroscience and the Child Study Center, School of Medicine, Yale University, New Haven, CT 06510 USA; 4 Connecticut Council on Problem Gambling, Wethersfield, CT 06109 USA; 5 Connecticut Mental Health Center, New Haven, CT 06519 USA; 6 Wu Tsai Institute, Yale University, New Haven, CT 06510 USA; 7 School of Psychological Sciences, Birkbeck, University of London, London, UK; 8 Social Determinants of Health Research Center, Research Institute for Prevention of Non- Communicable Diseases, Qazvin University of Medical Sciences, Qazvin, 3419759811 Iran; 9 Department of Nursing, School of Health and Welfare, Jönköping University, Gjuterigatan 5, Jönköping, 553 18 Sweden

**Keywords:** Adolescent, Confirmatory factor analysis, Gaming disorder, Measurement invariance, Psychometrics, Rasch, Insomnia, Psychological distress

## Abstract

**Background:**

Gaming Disorder (GD) was recently included by the World Health Organization (WHO) as a psychiatric condition in the eleventh revision of the International Classification of Diseases (ICD-11) and is a concern worldwide, including in Iran. Thus, based on the ICD-11 criteria, a Persian version of the Gaming Disorder Test (GDT) was developed to facilitate assessment of GD.

**Methods:**

The present study used classical test theory and Rasch analysis to examine the psychometric properties of the Persian GDT. Iranian adolescents (*n* = 3837; 2171 [56.6%] males; mean [SD] age = 16.02 [1.4] years) completed the GDT and other instruments assessing disordered gaming, psychological distress, and insomnia.

**Results:**

Overall, the psychometric properties of the Persian GDT were satisfactory based on classical test theory (i.e., confirmatory factor analysis corroborated the unidimensional structure of GDT) and Rasch analysis (i.e., fit statistics suggested that all items were embedded in the concept of GD). Moreover, the Persian GDT was found to be sex-invariant, displaying no items with substantial differential item functioning across sexes. Additionally, it was found that GD mediated associations between time spent gaming and measures of psychological distress and insomnia.

**Conclusion:**

The Persian GDT is a convenient and short instrument for assessing GD among Iranian adolescents. The mediating roles of GD in the associations between time spent gaming and psychological distress and between time spent gaming and insomnia suggest that targeting features of GD may reduce psychological distress and improve sleep for Iranian adolescents.

## Introduction

The importance of understanding and identifying disordered gaming has been acknowledged worldwide. In May 2013, the American Psychiatric Association (APA) published the fifth edition of the *Diagnostic and Statistical Manual of Mental Disorders* (DSM-5) that included nine diagnostic criteria for Internet Gaming Disorder (IGD) [1]. Following this, in May 2019, the World Health Organization (WHO) included Gaming Disorder (GD) as a disorder due to addictive behaviors in the eleventh revision of the *International Classification of Diseases* (ICD-11) [2]. Although the DSM-5 defines IGD with a focus on “online” gaming and the ICD-11 defines GD including both “online” and “offline” gaming, the current literature on IGD or GD sometimes considers both “online” and “offline” gaming in the context of disordered gaming, often with a focus on online gaming and particularly specific forms such as those involving multiple individuals across jurisdictions [3–5]. As such, gaming is a specific activity, while “online” and “offline” are different modes of gaming. Therefore, the impacts of online and offline gaming may or may not be substantial as compared to the main activity of gaming. Interestingly, both the APA and the WHO frameworks consider excessive gaming that elicits functional impairments an important aspect of the disordered gaming experience [6], with recent large-scale empirical research suggesting that within the APA framework, disordered gaming may translate to an average of 34.53 h of gaming a week whereas for the WHO framework, disordered gaming may correspond to an average of 40.13 h of gaming a week [7]. It should be noted that the high average hours of gaming a week seem higher than many other reports (e.g., the average weekly hours spent gaming were 7.71 for Americans, 12.39 h for Chinese, 10.16 h for Vietnamese, and 8.45 h regardless of ethnicity [8].

Although risk for disordered gaming may vary according to the type of device used for gaming [9], the definition of disordered gaming according to the DSM-5 and ICD-11 may help overcome inconsistent terminologies and unstandardized assessments adopted in the evaluation of disordered gaming [10–14]. In this regard, standardized psychometric tests aligning with the definitions proposed in the DSM-5 and/or ICD-11 have been developed and tested extensively recently [15]. However, most instruments have been developed using the APA-generated DSM-5 criteria for IGD (i.e., APA framework) instead of the more recent WHO-generated ICD-11 criteria for GD (i.e., WHO framework). Additionally, research has highlighted potential discrepancies in estimates of disordered gaming according to the two diagnostic frameworks [16, 17], an issue warranting further investigation.

Having standardized instruments developed using the well-defined WHO framework for GD is important as the ICD-11 is used clinically for many purposes globally. Furthermore, the development of sound psychometric tests based on the WHO framework for GD complements the existing psychometric tests for disordered gaming that have been developed adopting the APA framework [15]. Specifically, psychometric tests developed using the APA framework may have an advantage of providing comprehensive information on different aspects of disordered gaming. In contrast, psychometric tests developed using the WHO framework may have an advantage of assessing disordered gaming more parsimoniously via brief assessment [18–20]. Currently, there are several available psychometric tests assessing GD using the WHO framework, such as the Gaming Disorder Test (GDT) [21] and the Gaming Disorder Scale for Adolescents [22, 23].

In the present study, the GDT was translated into Persian and tested for its psychometric properties. The reasons for choosing the GDT instead of the Gaming Disorder Scale for Adolescents were as follows. First, although both instruments are brief, the GDT is more concise as it contains only four items covering all key criteria for GD while the Gaming Disorder Scale for Adolescents contains 10 items [21–23]. Second, the GDT includes interference in one or more domains of functioning in addition to the other three criteria described in the ICD-11; please see the *Measures* section for additional information [21]. Third, the GDT may apply to multiple developmental groups, including adolescents. Fourth, the GDT appears to have more cross-cultural support as it has been translated and validated in several cultural contexts and languages, including Bengali [24], Chinese (Mandarin), English, German, Malay [18], Turkish, Polish [25], and European Spanish [16, 21, 26, 27].

Because the GDT has never been translated into Persian, the psychometric properties of the Persian GDT remain unclear. The Persian-speaking population is large, estimated at 120 million people worldwide [28]. In addition, GD is relevant to Persian-speaking populations [29–31]. Indeed, a high prevalence estimate (over 20%) for GD has been reported for Iran, a major Persian-speaking country [8]. Therefore, translating and validating the GDT into Persian could have substantial public health implications.

In addition to exploring the psychometric properties of the GDT within a Persian cultural context, it is important to know how time spent gaming may relate to GD and how GD may relate to health measures, such as psychological distress and poor sleep. Excessive time spent gaming relates to disordered gaming [32]; however, ICD-11-defined GD includes a focus on negative psychosocial health consequences of gaming [33]. More severe GD has previously been associated with greater psychological distress [34–37], with data suggesting that GD leads to psychological distress over time in youth [34]. In addition, prior studies have shown that GD could lead to poor sleep, sleep disturbances, and insomnia [38, 39]. Therefore, assessing associations between GDT scores and psychological distress and sleep measures could provide additional insights for healthcare providers to intervene in multiple related health domains.

Given the above information, we hypothesized that Iranian adolescents who engaged more frequently in gaming would have more severe GD, and this would in turn relate to greater levels of psychological distress. Similarly, GD has been linked to insomnia among youth [40], with specific mechanisms (e.g., poor control over gaming, light emission from screens, and melatonin dysregulation) proposed [41]. Thus, we hypothesized a similar relationship between GD severity and insomnia in Iranian youth.

The present study aimed to develop the Persian GDT and test in a large sample of adolescents in Iran to assess its psychometric properties using two psychometric theories (i.e., classical test theory and Rasch analysis). Moreover, the present study proposed two mediation models that investigated the extent to which GD may mediate associations between time spent gaming and health outcomes (i.e., psychological distress and insomnia).

## Method

### Participants and procedure

Participants were adolescents studying in high schools in Qazvin, Iran. Adolescents were randomly recruited from 50 high schools in Qazvin using a two-stage sampling technique. In the first stage, 12 high schools were randomly selected from all high schools in Qazvin. In the second stage, all adolescents were then invited to participate in the study. All adolescents and their parents needed to read and provide signed informed consent before participation. The study was approved by the Ethics Committee of Qazvin University of Medical Sciences (no. IR.QUMS.REC.1401.158) and the Organization for Education in Qazvin.

### Translating the Gaming Disorder Test (GDT) into Persian

To translate the GDT into Persian, Beaton’s guidelines [42] were adopted. First, the English version of the GDT was translated into Persian by two independent bilingual translators (forward translation). Subsequently, both translated versions were synthesized into an interim Persian version in a session. The interim version was then translated back into the English by two independent bilingual translators who were blinded to the original English version. All translated versions were then consolidated during an expert committee session. The committee members (including a psychologist, school nurse, and psychiatrist) reviewed all translations to achieve equivalence between the English and Persian versions. The interim Persian version was then piloted on 39 adolescents. The final version was then administrated to 3837 adolescents to measure the psychometric properties of the Persian GDT.

### Measures

#### Background information

Background information was collected from participants including their age (reported in years), sex (reported as male or female), and time spent gaming per week (reported in hours).

#### Gaming Disorder Test (GDT)

The GDT [21] contains four items developed based on the WHO framework for GD as defined initially in the ICD-11 [42]. Specifically, the four items reflect (i) impaired control; (ii) increasing priority; (iii) continuation despite negative consequences; and, (iv) experience of significant life problems due to gaming. The GDT items were rated on a five-point Likert scale (1 = never to 5 = very often), and higher scores reflect more severe disordered gaming. The GDT was found to have satisfactory psychometric properties in its English and Chinese versions [21] and in all subsequent psychometric validation studies conducted in other languages.

#### Depression, Anxiety, Stress Scale (DASS-21)

The DASS-21 [43] contains 21 items assessing three types of psychological distress: depression, anxiety, and stress [44]. The DASS-21 items are rated on a four-point Likert scale (1 = never to 4 = almost all the time), and higher scores reflect higher levels of psychological distress. The DASS-21 has demonstrated satisfactory psychometric properties across different languages [45–48], including Persian [49].

#### Insomnia Severity Index (ISI)

The ISI [50] contains seven items developed using diagnostic criteria proposed in the fourth edition of the DSM (DSM-IV) and the International Classification of Sleep Disorders (ICSD) for insomnia [51]. The ISI items are rated on a five-point Likert scale (0 = no problem to 4 = very severe problem), and higher scores reflect higher levels of insomnia. The ISI has demonstrated satisfactory psychometric properties across different languages [52, 53], including Persian [54].

### Statistical analysis

Background information was analyzed using descriptive statistics. Then, two types of psychometric testing were used to examine the properties of the Persian GDT. Psychometric testing using classical test theory included internal consistency and confirmatory factor analysis (CFA), with a diagonally weighted least squares estimator. Psychometric testing using Rasch analysis included separation reliability, separation index, and unidimensional testing via variance of standardized residuals. Moreover, measurement invariance via CFA and differential item functioning (DIF) via Rasch were used to understand if different subgroups interpreted the GDT items and structure differently. Lastly, mediation models examined whether severity of GD mediated associations between gaming time and psychological distress and time spent gaming and insomnia.

Regarding the thresholds used for testing the psychometric properties of the Persian GDT in the classical test theory, a Cronbach’s α > 0.7 indicated good internal consistency [55]; a comparative fit index (CFI) > 0.9 together with a Tucker-Lewis index (TLI) > 0.9, root mean square error of approximation (RMSEA) < 0.08, and standardized root mean square residual (SRMR) < 0.08 in the CFA indicated unidimensionality [56]. Moreover, the CFI and TLI both indicate whether the proposed model (i.e., a one-factor structure model of GDT in the present study) outperformed the worst model (i.e., a null model that assumes all GDT items are not correlated). The difference between the CFI and TLI is that they provide different penalties on complex models. The RMSEA indicates whether the proposed model is convergently fit to the analyzed data with the consideration of model complexity. The SRMR indicates the differences between observed correlations among the items and the correlations in the proposed model without considering model complexity. In Rasch analysis, infit and outfit mean square (MnSq) between 0.5 and 1.5 indicated good fit of the item [57]; item and person separation reliability > 0.7 indicated good reliability in reproducibility of the Persian GDT items [58]; item and person separation indices > 2 indicated good discrimination in identifying different levels of GD [58]; the first contrast of the eigen value in the unexplained variance derived from Rasch standardized residuals < 1.5 indicated unidimensionality of the Persian GDT [59].

For measurement invariance, nested models across sexes (i.e., male vs. female) and across time spent gaming using average hours spent gaming in the present sample (i.e., ≤ 19.5 hours per week *vs.* >19.5 hours per week [19.5 hours is the average hours spent gaming in the present sample]) in the CFA (so-called multigroup CFA) were constructed in four levels: (i) a configural model that did not constrain any factor loadings, item intercepts, and residuals across subgroups; (ii) a metric invariance model that constrained factor loadings across subgroups; (iii) a scalar invariance model that constrained factor loadings and item intercepts across subgroups; and (iv) a strict invariance model that constrained factor loadings, item intercepts, and residuals across subgroups [60]. The fit indices of a more constrained model were used to deduct those of a less constrained model (e.g., the CFI of the scalar invariance model deducted the CFI of the metric invariance model). Measurement invariance was supported when ∆CFI > -0.01, ∆SRMR < 0.015, and ∆RMSEA < 0.015 [61]. For DIF, the differences of difficulties between subgroups were calculated to indicate DIF contrasts, of which < 0.5 indicated acceptable DIF [62]. A latent profile analysis (LPA) was conducted on all 4 items of the Persian GDT to identify different levels of GD according to their underlying latent structure of the GDT. Several indices were used to evaluate the LPA model: Akaike information criterion (AIC), Bayesian information criteria (BIC), entropy, and sample-size-adjusted Bayesian information criterion (SSABIC). Mplus (version 7.0) was used for the LPA.

Hayes’ Process macro (Model 4) [63] was used to examine if GD mediated associations between gaming time and psychological distress and between gaming time and insomnia level. A bootstrapping method with 5000 resamples was used to examine if the mediations were significant. When the 95% upper-limit confidence interval (ULCI) and lower-limit confidence interval (LLCI) did not cover 0, the significance of GD as a mediator was supported [64].

## Results

Among the 3837 adolescents included in the final sample, over half were male (n = 2171; 56.6%). The mean age was 16.02 years (SD = 1.40 years). On average, participants spent 19.50 (SD = 14.30) hours gaming per week. Moreover, 1268 participants (33.04%) spent 20 hours or more gaming. Detailed information on psychological distress and insomnia is presented in Table 1. Table 1 also reports mean (SD) GDT scores. There was evidence of some floor effects for the four GDT items. The number (percentage) of participants scoring 1 for GDT item 1 was 1323 (34.5%), 1847 (48.1%) for GDT item 2, 1468 (38.3%) for GDT item 3, and 1869 (48.7%) for GDT item 4. In addition, the LPA results showed that the 4 GDT items can be categorized into 2 groups with high and low risk of GD (AIC = 43680.133, BIC = 43761.411, SSABIC = 43720.103, entropy = 0.852, and LMR test = 321.016). Adolescents at high (38.1%) and low risk (61.9%) of GD did not differ from each other in age (mean [SD] of high-risk group: 16.03 [1.50]; mean [SD] of low-risk group: 16.0 [1.39]) and sex categories (n [%] of male high-risk group: 1368 [57.6%]; n [%] of male low-risk group: 802 [54.8%]). Moreover, adolescents at high risk of GD reported significantly higher distress (mean [SD] of high-risk group: 30.09 [9.49]; mean [SD] of low-risk group: 24.86 [7.68] in DASS-21), insomnia (mean [SD] of high-risk group: 11.54 [3.85]; mean [SD] of low-risk group: 7.88 [2.25] in ISI), time spent gaming (mean [SD] of high-risk group: 9.20 [6.22]; mean [SD] of low-risk group: 7.69 [5.43] in hours per week) (mean = 9.20, SD = 6.22), and GDT scores (mean [SD] of high-risk group: 10.08 [3.76]; mean [SD] of low-risk group: 5.42 [2.13] in GDT) (*p*-values < 0.001).

**Table 1:** Participant characteristics and GDT scores (N = 3837)

	Mean ± SD or n (%)
Age (Years)	16.02 ± 1.40
Sex (Male)	2171 (56.6%)
Score on the DASS-21, depression^a^	8.5 ± 4.1
Score on the DASS-21, anxiety^a^	9.6 ± 4.0
Score on the DASS-21, stress^a^	8.7 ± 4.5
Score on the Insomnia Severity Index^b^	10.48 ± 5.12
Weekly time spent gaming on the internet (Hours)	19.50 ± 14.30
Score on GDT-1	2.12 ± 1.09
Score on GDT-2	1.81 ± 1.01
Score on GDT-3	2.22 ± 1.23
Score on GDT-4	1.99 ± 1.56
GDT total score	8.27 ± 4.08

^a^ Measured using the Depression Anxiety Stress Scales (DASS-21)

^b^ Measured using Insomnia Severity Index

GDT = Gaming Disorder Test

Psychometric properties of the Persian GDT at the item level were satisfactory as the factor loadings (ranging from 0.684 to 0.749), corrected item to total correlations (ranging from 0.499 to 0.557), and MnSq (infit = 0.92 to 1.06; outfit = 0.90 to 1.03) all fit with recommended thresholds. Moreover, no substantial DIF was identified for any GDT item across sexes (DIF contrast = -0.20 to 0.21) or across time spent gaming (DIF contrast = -0.21 to 0.19) (see Table 2).

**Table 2:** Psychometric properties of the Gaming Disorder Test (GDT) at the item level

Item #	Classical test theory analyses		Rasch analyses					
	Factor loading^a^	Item-total correlation	Infit MnSq	Outfit MnSq	Discrimination	Difficulty	DIF contrasts across sexes^cd^	DIF contrasts across gaming-time groups^ce^
GDT-1	0.684	0.507	0.96	0.96	0.99	-0.27	-0.03	-0.04
GDT-2	0.713	0.557	0.92	0.90	1.07	0.34	0.21	0.19
GDT-3	0.709	0.499	1.05	1.03	0.94	-0.23	0.10	-0.21
GDT-4	0.749	0.533	1.06	1.00	1.01	0.16	-0.20	0.10

^a^ Based on confirmatory factor analysis

^b^ Using Pearson correlation

^c^ DIF = differential item functioning; DIF contrast > 0.5 indicates substantial DIF

^d^ DIF contrast across sexes = Difficulty for females-Difficulty for males

^e^ DIF contrast across time on gaming = Difficulty for participants with median weekly hours or below spent gaming (i.e., < 19.5 h)-Difficulty for participants with above median weekly hours spent gaming (i.e., ≥ 19.5 h)

MnSq = mean square error; DIF = differential item functioning

Regarding the GDT’s psychometric properties at the scale level, a unidimensional structure was fully supported for the GDT by both CFA and Rasch results. In the CFA results, all fit indices were satisfactory (i.e., χ^2^ = 1.810; df = 2; CFI = 1.000; TLI = 1.000; RMSEA = 0.001; and SRMR = 0.009, see Table 3). Moreover, the df larger than χ^2^ values explains the satisfactory fit of the CFI and RMSEA. In the Rasch results, a strong contrast of eigenvalue explained by the Persian GDT was extracted (3.75; 48.4%), and the first contrast on unexplained variance had a low eigenvalue (1.40; 18.2%) (see Table 4). Additionally, internal consistency (α = 0.73), separation reliability (item separation reliability = 0.99; person separation reliability = 0.78), and separation index (item separation index = 11.63; person separation index = 2.61) were all satisfactory (see Table 3).

**Table 3:** Psychometric properties of the Gaming Disorder Test (GDT) at the scale level

Psychometric testing	Value	Suggested cutoff
Internal consistency (Cronbach’s α)	0.732	> 0.7
Confirmatory factor analysis		
χ^2^ (*df*)	1.810 (2)	Nonsignificant
Comparative fit index	1.000	> 0.9
Tucker-Lewis index	1.000	> 0.9
Root-mean square error of approximation	0.001	< 0.08
Standardized root mean square residual	0.009	< 0.08
Item separation reliability from Rasch	0.99	> 0.7
Item separation index from Rasch	11.63	> 2
Person separation reliability from Rasch	0.78	> 0.7
Person separation index from Rasch	2.61	> 2

**Table 4:** Variance of standardized residuals

	Eigenvalue	Observed (%)	Expected (%)
Total raw variance	7.75	100.0	100.0
Raw variance explained by measures	3.75	48.4	48.5
Raw variance explained by persons	1.90	24.5	24.5
Raw Variance explained by items	1.85	23.9	24.0
Raw unexplained variance (total)	4.00	51.6	51.5
Unexplained variance in the first contrast	1.40	18.2	35.2

The unidimensional structure of the Persian GDT was supported to be invariant across sexes and time spent gaming groups. Specifically, strict measurement invariance of the GDT unidimensional structure was supported across sexes by the fit indices of ∆CFI (0.000), ∆SRMR (range between 0.000 and 0.003), and ∆RMSEA (0.000). Similarly, the metric measurement invariance was confirmed across the time spent gaming groups: ∆CFI = 0.00, ∆SRMR = 0.005, and ∆RMSEA = 0.00. However, scalar and strict invariance were not supported by fit indices (scalar invariance: ∆CFI = -0.004, ∆SRMR = 0.005, and ∆RMSEA = 0.021; strict: ∆CFI = -0.02, ∆SRMR = 0.025, and ∆RMSEA = 0.024) (see Table 5).

**Table 5:** Measurement invariance across sexes and across gaming-time groups (based on weekly hours spent gaming on the internet) through confirmatory factor analysis

Model and comparisons	Fit statistics							
	χ^2^ (df)	∆χ^2^ (∆df)	CFI	∆CFI	SRMR	∆SRMR	RMSEA	∆RMSEA
**Sex**								
M1: Configural	3.118 (4)		1.000		0.011		0.001	
M2: Metric	3.689 (7)		1.000		0.012		0.001	
M3: Scalar	5.638 (10)		1.000		0.012		0.001	
M4: Strict	7.643 (14)		1.000		0.015		0.001	
M2 − M1		0.571 (3)		0.000		0.001		0
M3 − M2		1.949 (3)		0.000		0		0
M4 − M3		2.005 (4)		0.000		0.003		0
**Time spent gaming**								
M1: Configural	3.350 (4)		1.000		0.011		0.001	
M2: Metric	6.215 (7)		1.000		0.016		0.001	
M3: Scalar	19.688 (10) *		0.996		0.021		0.022	
M4: Strict	70.241 (14) *		0.976		0.046		0.046	
M2 − M1		2.865 (3)		0.000		0.005		0.000
M3 − M2		13.473 (3)		-0.004		0.005		0.021
M4 − M3		50.533 (4)		-0.020		0.025		0.024

*p < 0.05

Additionally, GD mediated the relationship between time spent gaming and psychological distress (unstandardized coefficient = 0.071; bootstrapping SE = 0.011; LLCI = 0.052; ULCI = 0.093). GD also mediated the relationship between time spent gaming and insomnia (unstandardized coefficient = 0.058; bootstrapping SE = 0.003; LLCI = 0.050; ULCI = 0.065) (see Table 6).

**Table 6:** Models of the effect of time spent gaming on psychological distress and insomnia with Gaming Disorder (GD) tested as a mediator

	Unstand. Coeff.	SE or (Bootstrapping SE)	t-value or (Bootstrapping LLCI)	*p*-value or (Bootstrapping ULCI)
**Psychological distress as outcome**				
Total effect of time spent gaming on psychological distress	0.262	0.010	25.791	< 0.001
Direct effect of time spent gaming on psychological distress	0.191	0.010	17.685	< 0.001
Direct effect of time spent gaming on Gaming Disorder Test (GDT)	0.088	0.003	27.908	< 0.001
Indirect effect of time spent gaming on psychological distress via GDT	0.071	(0.011)	(0.052)	(0.093)
**Insomnia as outcome**				
Total effect of time spent gaming on insomnia	0.073	0.003	23.666	< 0.001
Direct effect of time spent gaming on insomnia	0.016	0.002	6.204	< 0.001
Direct effect of GD on mediator	0.088	0.003	27.908	< 0.001
**GDT**				
Indirect effect of time spent gaming on insomnia via GDT	0.058	(0.003)	(0.050)	(0.065)

Note: Age, sex, paternal and maternal years of education were adjusted for the model

Unstand. Coeff.=unstandardized coefficient

LLCI = lower limit in 95% confidence interval

ULCI = upper limit in 95% confidence interval

Psychological distress was measured using the Depression, Anxiety, Stress Scale (DASS-21)

## Discussion

The Persian GDT demonstrated adequate psychometric properties in a large sample of Iranian adolescents. The linguistic validity of the Persian GDT was supported using a rigorous procedure following international guidelines [42]. The psychometric properties of the Persian GDT were deemed to be satisfactory using both classical test theory and Rasch analysis. Specifically, both classical test theory and Rasch analysis supported the unidimensionality of the Persian GDT, the fit of item properties in the GDT, the suitability of the GDT at a scale level, and measurement invariance and DIF of the GDT unidimensional structure and items across sex and time spent gaming groups. Moreover, GD mediated associations between time spent gaming and two health measures (psychological distress and insomnia).

The present study showed that the Persian GDT had comparable psychometric properties relative to other GDT language versions. Specifically, the Persian GDT demonstrated high internal consistency (α = 0.73) like the English (α = 0.87), simplified Chinese (α = 0.84) [21], traditional Chinese (α = 0.90) [31], Turkish (α = 0.84) [26, 65], and Spanish (α = 0.89) [27] versions. Furthermore, the unidimensionality of the Persian GDT was similar to findings from English, Chinese (both simplified and traditional written characters), Turkish, Spanish, and Malay versions [21, 26, 27, 31, 65]. The measurement invariance findings across sexes in the present study echoed findings from Maldonado-Murciano et al. [27]. Therefore, the obtained results are consistent with prior psychometric studies of the GDT regarding its appropriate reliability and unidimensionality. The present study extends prior findings in that the unidimensionality of the Persian GDT was supported across distinct groups representing different amounts of time spent gaming, which resonates with findings from mainland China [20].

The promising psychometric properties of the Persian GDT may stem from multiple factors. First, the original GDT was developed following the establishment of nomological validation and using ICD-11 criteria proposed within the WHO framework for disordered gaming [21]. Second, the translation procedure for the Persian GDT followed standardized international guidelines; that is, forward translation, reconciliation, back translation, expert panel review, cognitive interviewing, and pilot testing [42].

Furthermore, GD was also found to mediate associations between time spent gaming and two health measures: psychological distress and insomnia (where insomnia may be viewed as a proxy measure for poor sleep quality [66]). The mediating roles of GD were fully supported and in line with the existing literature. More specifically, excessive time spent gaming has been linked to the emergence of GD [32]. Additionally, associations between GD and psychological distress [34–36, 67, 68] and GD and sleep problems [38, 39, 69] have also been reported. Further, GD has been demonstrated to lead to psychological distress among youth longitudinally [34, 70, 71]. Similarly, GD has been proposed to lead to sleep disturbances, particularly among adolescents, through multiple mechanisms including poor control over gaming, light exposure relating to screen time, and melatonin dysregulation [72]. At biochemical and neurological levels, the amount of illumination from light exposure during gaming may suppress melatonin, disrupting sleep patterns [73]. Hence, individuals who spend more time gaming may be more likely to develop GD, which subsequently may lead to health issues including psychological distress and insomnia.

Major implications of the present findings include support for the usefulness of the Persian GDT in Persian-speaking settings when healthcare providers may want to assess gaming problems. As the Persian GDT contains only four items, it is likely to be an efficient assessment tool in identifying individuals with high risk of disordered gaming in busy clinical settings. In addition, the brevity of the GDT could help researchers to cost-effectively conduct studies assessing many different latent constructs in large samples. The usefulness of a brief instrument such as the GDT should be based on satisfactory psychometric properties. Therefore, the present study’s findings help justify the usefulness of the GDT with its advantages of being brief and concise.

Several potential study limitations warrant mention. First, the present study only used self-reported data, and biases related to self-report (e.g., social desirability bias, recall bias, and single-rater bias) cannot be excluded. Second, the present study did not examine the test-retest reliability of the Persian GDT. Future studies are warranted to investigate this feature. Third, the present sample was on average healthy in terms of psychosocial health (that is, their DASS-21 and ISI scores were at worst moderate). Therefore, the present findings may not generalize to a population with more severe mental health or sleep problems. Lastly, the present study used a cross-sectional design to examine the proposed mediation models, and future longitudinal studies are warranted.

## Conclusion

Taken together, the Persian GDT demonstrated satisfactory and unidimensional psychometric properties. It constitutes a brief and convenient instrument for assessing GD among Iranian adolescents. The Persian GDT psychometric properties were supported with respect to factor structure (via CFA and Rasch analysis), internal consistency (via Cronbach’s α), separation reliability and separation index (via Rasch analysis), measurement invariance (via CFA), and DIF (via Rasch analysis). Moreover, GD mediated associations between time spent gaming and psychological distress and time spent gaming and insomnia. In conclusion, the present study provides further evidence of the promising psychometric properties and suitability of the GDT to assess disordered gaming as per the WHO framework.

## Data Availability

Data are available from the corresponding author upon reasonable request.
